# Sevoflurane causes cognitive impairment by inducing iron deficiency and inhibiting the proliferation of neural precursor cells in infant mice

**DOI:** 10.1111/cns.14612

**Published:** 2024-02-09

**Authors:** Yong Zuo, Jinhong Xie, Xue Zhang, Anand Thirupathi, Xiaopeng Liu, Di Zhang, Jianhua Zhang, Zhenhua Shi

**Affiliations:** ^1^ Laboratory of Molecular Iron Metabolism, College of Life Science Hebei Normal University Shijiazhuang Hebei Province China; ^2^ Faculty of Sports Science Ningbo University Ningbo China; ^3^ The Second Affiliated Hospital of Hebei Medical University Shijiazhuang China

**Keywords:** cognitive impairment, iron metabolism, neurodevelopment, sevoflurane

## Abstract

**Aims:**

Numerous studies on animals have shown that exposure to general anesthetics in infant stage may cause neurocognitive impairment. However, the exact mechanism is not clear. The dysfunction of iron metabolism can cause neurodevelopmental disorders. Therefore, we investigated the effect of iron metabolism disorder induced by sevoflurane (Sev) on cognitive function and the proliferation of neural precursor cells (NPCs) and neural stem cells (NSCs) in infant mice.

**Methods:**

C57BL/6 mice of postnatal day 14 and neural stem cells NE4C were treated with 2% Sev for 6 h. We used the Morris water maze (MWM) to test the cognitive function of infant mice. The proliferation of NPCs was measured using bromodeoxyuridine (BrdU) label and their markers Ki67 and Pax6 in infant brain tissues 12 h after anesthesia. Meanwhile, we used immunohistochemical stain, immunofluorescence assay, western blot, and flow cytometer to evaluate the myelinogenesis, iron levels, and cell proliferation in cortex and hippocampus or in NE4C cells.

**Results:**

The results showed that Sev significantly caused cognitive deficiency in infant mice. Further, we found that Sev inhibited oligodendrocytes proliferation and myelinogenesis by decreasing MBP and CC‐1 expression and iron levels. Meanwhile, Sev also induced the iron deficiency in neurons and NSCs by downregulating FtH and FtL expression and upregulating the TfR1 expression in the cortex and hippocampus, which dramatically suppressed the proliferation of NSCs and NPCs as indicated by decreasing the colocalization of Pax6^+^ and BrdU^+^ cells, and caused the decrease in the number of neurons. Interestingly, iron supplementation before anesthesia significantly improved iron deficiency in cortex and hippocampus and cognitive deficiency induced by Sev in infant mice. Iron therapy inhibited the decrease of MBP expression, iron levels in neurons and oligodendrocytes, and DNA synthesis of Pax6+ cells in hippocampus induced by Sev. Meanwhile, the number of neurons was partially recovered in hippocampus.

**Conclusion:**

The results from the present study demonstrated that Sev‐induced iron deficiency might be a new mechanism of cognitive impairment caused by inhaled anesthetics in infant mice. Iron supplementation before anesthesia is an effective strategy to prevent cognitive impairment caused by Sev in infants.

## Graphical abstract

Sevoflurane inhibits the proliferation of neural precursor cells by causing iron deficiency in infant mice which further inducescognitive dysfunction. Iron supplementation before anesthesia is an effective strategy to prevent cognitive impairment caused by Sev in infants.

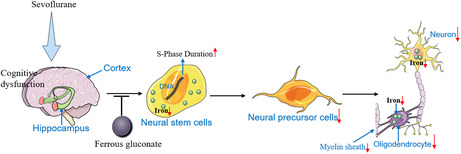

## 1 INTRODUCTION

Each year millions of children under 3 years of age require general anesthesia for various reasons.^1^ Anesthetics are drugs that are used during surgery. Despite their proven safety, the neurotoxicity induced by anesthetics, especially in infants or young children, has raised wide concerns.^2^, ^3^ Therefore, the US Food and Drug Administration (FDA) released a new warning regarding the use of anesthetics in children under 3 years of age.^4^

Sev is a commonly used anesthetic in pediatric surgery. It was reported that Sev could cause the long‐term diminution of cognition and regional volumetric alterations in brain structure in early childhood which was associated with brain development.^1^, ^5^ Our previous and other studies showed that Sev could inhibit the myelin sheath formation when the fetus or young mice were exposed to Sev, inducing cognitive dysfunction.^6^, ^7^ Neonatal exposure to anesthesia leads to cognitive deficits in old age.^8^ In addition, exposure of young animals, including both rodents and primates (including humans), to general anesthesia causes nerve cell apoptosis,^8^, ^9^ which further affects brain development.

Iron is one of the essential trace elements for human beings which is involved in many metabolic processes in the central nervous system, including energy production, DNA synthesis, and oxygen transport.^10^, ^11^ Hence, maintaining iron homeostasis plays an important role in infant brain development. Ferritin is a type of iron storage protein that includes two subunits: heavy‐chain ferritin (FtH) and light‐chain ferritin (FtL). Transferrin receptor1 (TfR1) contributed to the iron ions in cells. The expressions of ferritin and TfR1 are regulated by cellular iron concentration via the IRP/IRE system. When cellular iron levels increased, ferritin expression upregulated and TfR1 expression downregulated. On the contrary, ferritin expression downregulated and TfR1 expression upregulated. Our previous experiments showed that Sev could cause the iron homeostasis disorder to induce neurotoxicity in elder mice, and higher iron levels further affected the function of mitochondrial and ATP production.^12^, ^13^

Some studies showed that Sev contributed to neurotoxicity by inducing apoptosis and inflammation.^14^, ^15^ NSCs and NPCs have the potential to differentiate into neurons, astrocytes, and oligodendrocytes to produce a large number of mature brain cells. A recent study showed that Sev also affected the neurogenesis and differentiation to relieve brain development by inhibiting cell cycle and DNA methylation of NSCs in fetal or young mice.^16^, ^17^

These findings suggested a functional connection between NSCs or neuron damage and Sev. However, it remains largely unknown how Sev could affect the NSCs growth and differentiation and further damage neuron function, inducing cognitive impairment in young mice. Considering the role of iron in growth and differentiation, we tested the hypothesis that Sev could cause a cognitive impairment via dysfunction of iron metabolism in NSCs and neurons. We found that Sev caused the iron deficiency in NSCs, oligodendrocytes, and neurons, which inhibited neural development, inducing cognitive impairment. Iron supplementation before anesthesia is an effective strategy to prevent cognitive impairment caused by Sev.

## 2 MATERIALS AND METHODS

### 2.1 Experimental design, mice anesthesia, BrdU treatment, and iron therapy

**FIGURE 1:**
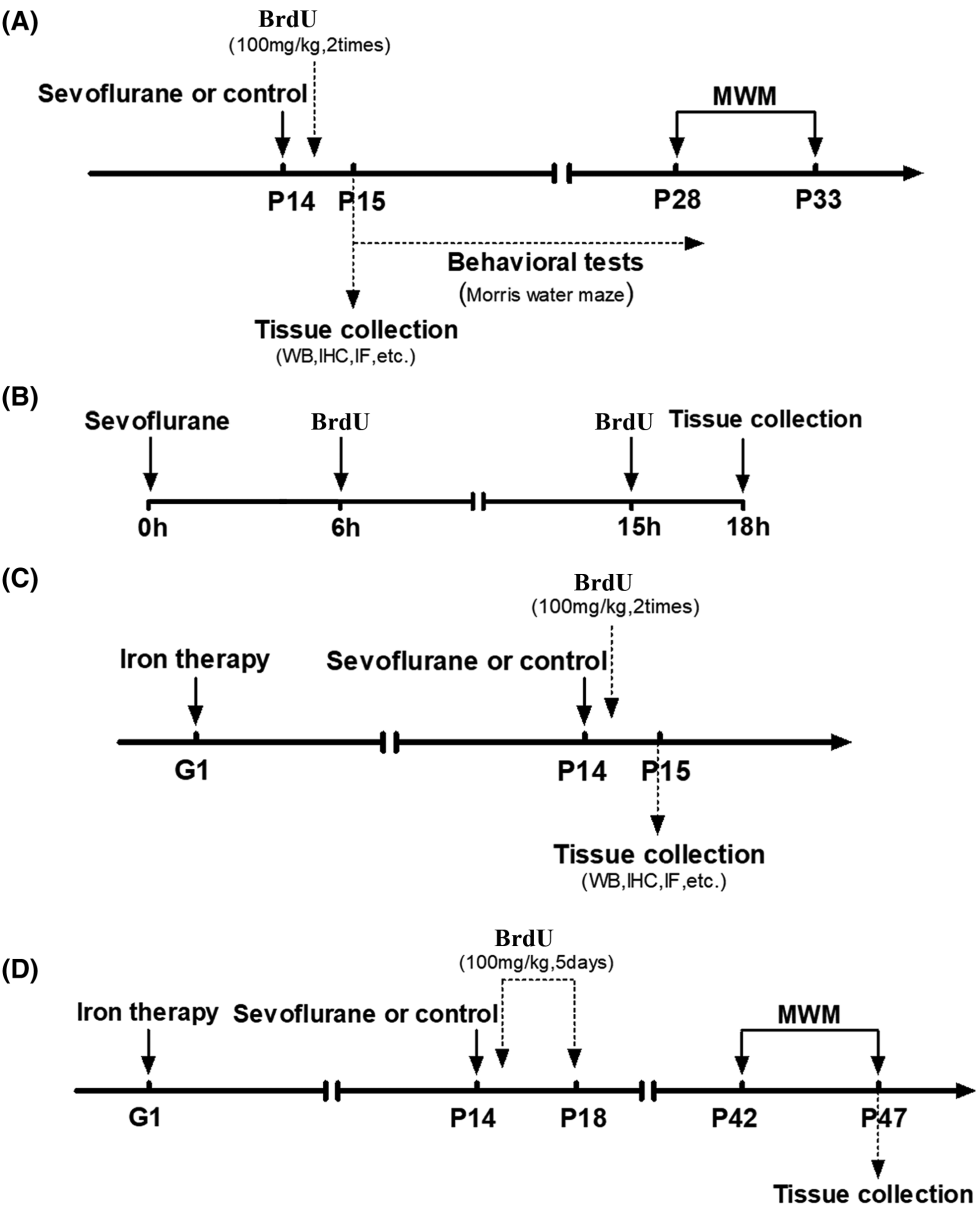
Schematic diagram of experimental design. (A) The mice of postnatal day 14 (P14) were treated with Sev for 6 h. Twelve hours after the anesthesia, the mice were divided into two groups randomly. One group of mice was used to evaluate the cognitive function by MWM when the mice of postnatal day 28. Another group of mice was harvested to assay the protein expression levels. (B) The mice of postnatal day 14 (P14) were treated with Sev for 6 h. After anesthesia, the mice were immediately treated with BrdU (100 mg/kg, intraperitoneal injection). After 9 h, the mice were treated with BrdU again. After 3 h, the brain tissues were harvested to run a follow‐up experiment. (C) On their first day of pregnancy (G1), the mice were fed with drinking water‐containing gluconate solution (0.1 mg/mL). The next step was the same as (A) and (B). (D) It took 28 days for NSCs to differentiate into other nerve cells. To evaluate the effect of Sev on the differentiation of NSCs, the iron and BrdU treatments were as same as (B) and (C). Twenty‐eight days after Sev treatment, MWM tested the cognitive function of mice.

All animal experiments were performed in accordance with ethical standards, and the procedures were approved by the Hebei normal university ethics committee. P14 days C57BL/6 male mice were fed in stainless steel rust‐free cages at 22–24°C and a 12‐h light/dark cycle and were provided free access to food and distilled water. The mice were assigned into five groups: control group, Sev treatment group 1 (14 days after anesthesia), Sev treatment group 2 (28 days after anesthesia), iron treatment group, and Sev + iron treatment group. Since the mice of P14 days group could only rely on breast milk for nutrition, we treated pregnant mice with iron supplementation from the first day of pregnancy in order to ensure adequate brain iron levels in the infant mice. Anesthesia treatment was according to our previous report.^6^ Briefly, the mice in the anesthesia groups were placed in an anesthetic induction chamber filled with Sev (3%) for approximately 5 min until they became unconscious. The mice were then removed and attached to one of the nose cones of the anesthetizing apparatus in order to be exposed to the same amount of anesthetic. The mice were treated with gases of 3% Sev and 40% oxygen within the anesthetic chamber for 6 h and were continuously monitored (Ohmeda Excel 210 SE anesthetic machine, Datex Instrumentarium Corp., Helsinki, Finland). For the iron therapy, the maternal drinking water of the offspring mice contained 10 mg/L ferrous gluconate, and the infant mice 14 days after birth were treated with 3% Sev for 6 h. The molecular mechanism was assessed on the 15th day after birth. The water maze and the effects of Sev on the proliferation of progenitor cells were detected after 28 days of Sev treatment. BrdU labeling, behavioral tests, and iron therapy of mice are shown in Figure 1.

### 2.2 Cell culture and anesthesia

NE4C cells, which were neuron stem cells from mice, were grown in MEM supplemented with 10% fetal bovine serum, 100 U/mL of penicillin, and 100 mg/mL of streptomycin. Cells were maintained at 37°C in a humidified 5% CO_2_ /95% air incubator. To investigate the effect of Sev on the NE4C cells, when the cells were in the logarithmic phase, we treated the NE4C cells with 2% Sev for 6 h in an anesthetic chamber in a carbon dioxide incubator with 5% CO_2_ at 37°C. The cells were cultured for another 4 h and then were harvested to be assayed.

### 2.3 Morris water maze (MWM) test

MWM test was done according to our previous report.^6^ Briefly, for mice in the control group, each mouse was given swimming training one time a day for 6 days. For Sev group mice, the mice were first anesthetized, and then they were given the MWM test. Then, these mice were given swimming training one time a day for 6 days. On the sixth day, the platform was removed to test the times of crossing the platform. For each mouse, the escape latency was recorded as a maximum of 120 s.

### 2.4 Immunohistochemistry studies and immunofluorescence assay

For the immunohistochemistry assay, the brains were carefully dissected, removed, post‐fixed, and then transferred to 30% sucrose for 2 days. The coronal brain sections were cut and frozen with a thickness of 15 μm, and then sections were washed with 0.01 M PBS three times for 5 min each time. The sections were incubated with 3% H_2_O_2_ for 20 min and then were washed with 0.01 M PBS three times for 5 min each time. The sections were then incubated with goat serum at 37°C for 60 min. The samples were incubated overnight at 4°C with the rabbit polyclonal anti‐MBP antibody (Cat. No. 10458‐1‐AP; Proteintech, Wuhan, China) and anti‐CC‐1 antibody (1:250; Cat. No. ab16794; Abcam, Massachusetts, USA). Next, they were incubated with biotinylated secondary goat anti‐rabbit IgG at 37°C for 1 h. After being washed with PBS three times for 5 min each time, the sections were incubated in horseradish peroxidase (HRP)‐labeled streptavidin reagent for 1 h at room temperature and then were stained using a DAB kit (SK‐4100; VECTOR, Kronshagen, Germany) for 40 s. Finally, the sections were dehydrated and mounted. Images were captured using a ZEISS LSM710 (LSM710; ZEISS, Germany). ImageJ was used to analyze the ratio of MBP‐positive signal area to total area as the expression level of MBP.

For the immunofluorescence assay, mouse monoclonal anti‐NeuN antibody (1:200; Cat. No. ab104224; Abcam, Massachusetts, USA), rabbit monoclonal anti‐FtL (1:400; Cat. No. ab109373; Abcam, Massachusetts, USA), and rabbit monoclonal anti‐FtH (1:400; Cat. No. ab183781; Abcam, Massachusetts, USA) were used as primary antibodies. FITC‐conjugated and rhodamine‐conjugated secondary antibodies were used. Images were captured using a ZEISS LSM710 (LSM710; ZEISS, Germany).

### 2.5 Transmission electron microscopy (TEM)

TEM was carried out according to Min Liu's method (Liu et al., 2018). Briefly, hippocampus tissues were dissected from mice treated with Sev and then the samples were fixed in 2.5% glutaraldehyde/2% paraformaldehyde (pH 7.2) overnight at 4°C. The samples treated with osmium tetroxide were dehydrated with different concentrations of ethanol and embedded with a mixture of resin and propylene oxide. Finally, tissues were in a 65°C oven for 48 h to polymerize, and then the samples were sectioned at 100 nm, lifted onto 3‐mm copper grids, and stained for 30 min in 1.5% aqueous uranyl acetate for 10 min in lead citrate. The sample sections were dried and viewed on the Hitachi H7000 TEM. Both the inner diameter and out diameter of the axons were quantified.

### 2.6 Perl's iron staining

The brain slices were washed with 0.01 mol/L PBS (pH 7.4) three times, for 5 min each, and then the slices were incubated with 3% H_2_O_2_ for 20 min. The slices were washed with 0.01 mol/L PBS (pH 7.4) three times, 5 min each again. The slices were stained using Perl's dye liquor for 8 h and then were washed with 0.01 mol/L PBS (pH 7.4) three times, 5 min each. Finally, the slices were strengthened using dyeing with DAB–H_2_O_2_ solution and then were washed with double‐distilled water for 30 min. After being washed with a gradient of ethanol and xylene, the slices were packaged with neutral gum, and the images were taken with the microscope (ZEISS Axio imager).

### 2.7 Western blot analysis

The hippocampal and cortex tissues were homogenized in RIPA buffer followed by centrifugation at 12,000 *g* for 20 min at 4°C. The supernatant‐containing proteins were collected and their content was measured using a protein quantification kit (KangWei, Beijing, China). The samples were resolved by 8–12% of SDS‐PAGE, respectively, and then transferred to nitrocellulose membranes (Millipore, Bedford, MA, USA). The target proteins FtH (ab109373; Abcam, USA), FtL (ab183781; Abcam, USA), NeuN (ab104224; Abcam, USA), Ki67 (ab15580; Abcam, USA), and CC‐1 (ab16794; Abcam, USA), MBP (10458‐1‐AP; Proteintech, Wuhan, China), TfR1 (136,800; ThermoFisher Scientific, USA), Pax6 (862002; Biolegend, USA), IBA1 (GTX632426), and GFAP (SAB5201104; Millipore, USA) were detected by their primary antibodies. The relative expression quantity of proteins was normalized to that of β‐actin (CW0096M; KangWei, Beijing, China).

### 2.8 Statistic analysis

In this study, GraphPad Software's Prism 7 (GraphPad Software, USA) was used to analyze the data as mean ± SEM. All western blotting data were obtained based on gray values using ImageJ Software, and the ratio of target protein gray value to actin gray value in the control group was 1. The normality of the data was analyzed using the Shapiro–Wilk test. For data with normal distribution, comparisons were performed using the unpaired t‐test or multiple *t*‐tests. Continuous variables data with nonnormal distributions were analyzed by the Mann–Whitney test. All data are expressed as mean ± SD. For the outcome of neurobehavioral changes, 90 infant mice were selected and divided into five groups: control group, Sev treatment group 1, Sev treatment group 2, iron treatment group, and Sev + iron treatment group, with 15 mice in each group. Six mice per group were used to test the relative protein expression by western blot, and three mice per group were for immunostaining studies, Perl's staining, and immunohistochemical analysis, respectively. In the MWM test, two‐way ANOVA and repeated measurements were used to analyze differences in learning curves (based on escape latency) among the control group, Sev treatment group 1, Sev treatment group 2, iron treatment group, and Sev + iron treatment group. The Mann–Whitney test or Kruskal–Wallis test was used to determine the difference in the number of platform crossings between the control and Sev groups or other groups. The difference in the two‐group comparison was determined by using a Student's *t*‐test when the data passed normality testing. One‐way ANOVA and individual Student's *t*‐test with Bonferroni correction were used to determine differences among groups in FtH, FtL, TfR1, MBP, CC‐1, NeuN, GFAP, IBA1, and iron content. A probability level of 95% (*p* < 0.05) was considered statistically significant, and significance testing was two‐tailed. Finally, it is important to note that adjusted *p*‐values of Bonferroni correction were calculated by dividing the accurate *p*‐values by experimental size, and adjusted *p*‐values were reported.

## 3 RESULTS

### 3.1 Sev caused cognitive deficiency and a decrease in the expression of myelin basic protein (MBP) and number of oligodendrocyte and iron levels of oligodendrocytes

**FIGURE 2:**
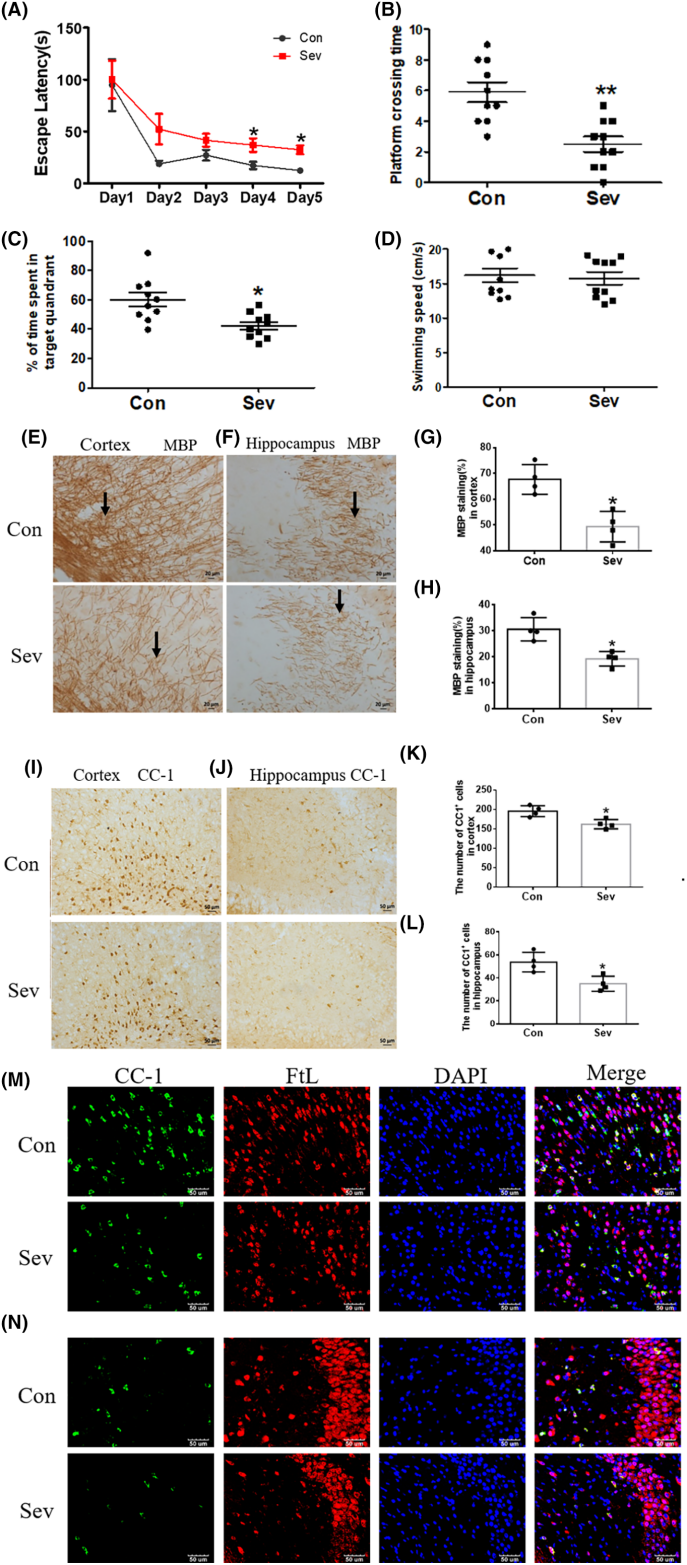
The effects of Sev on cognitive function, MBP and CC‐1 expression in cortex and hippocampus, and FtL expression in oligodendrocytes. The mice were treated with Sev as described in the ‘Section 2’. The cognitive function and swimming speed were detected by the MWM test (A–D). The results were calculated as the escape latency (sec.) and platform crossing time ± SD (*n* = 10 mice/group). (E–H) The expression of MBP in cortex and hippocampus. (I–L) The number of CC‐1^+^ cells in cortex and hippocampus using immunohistochemistry (*n* = 4 mice/group). (M) Expression of FtL in oligodendrocytes of cortex. (N) Expression of FtL in oligodendrocytes of hippocampus. Nuclei were counterstained with DAPI. Two‐way ANOVA: (A). Mann–Whitney test: (B–D). Student's *t*‐test: (G, H, K, L). *p < 0.05.

We treated P14 mice with 3% Sev for 6 h. Cognitive function of the mice was detected by MWM on the 14th day after anesthesia. The results showed that the escape latency of Sev group increased (32.5 ± 7 vs. 12.4 ± 4 s, *p* = 0.016, *F* = 30, *n* = 15, Figure 2A), but platform crossing time (5.6 ± 1.5 vs. 2.5 ± 1, *p* = 0.0483, Figure 2B) and time spent in target quadrant (61.2 ± 10% vs. 44.3 ± 5%, *p* = 0.0421, Figure 2C) decreased, indicating that Sev caused the cognitive deficiency in infant mice. However, Sev did not affect the swimming speed of mice (*p* = 0.647, Figure 2D). Myelin sheath played an important role in learning and memory.^6^ MBP, which mainly arose from the gene in the oligodendrocyte, was essential to the formation of the multilamellar myelin sheath of the mammalian central nervous system (CNS),^18^ and CC‐1 was an important marker protein for oligodendrocyte labeling.^19^

In order to investigate the relationship between cognitive deficiency and myelin sheath formation, as well as the number of oligodendrocytes, we detected the expression of MBP and the number of CC‐1^+^ cells. We found that Sev dramatically downregulated the MBP expression in cortex (68.60% ± 4% vs. 49.9 ± 6%, *p* = 0.0298, Figure 2E,G) and hippocampus (30.8% ± 3% vs. 19.1% ± 2%, *p* = 0.0335, Figure 2F,H). Meanwhile, Sev also decreased the number of CC‐1^+^ cells in cortex (198 ± 17 vs. 162 ± 13, *p* = 0.0473, Figure 2I,K) and hippocampus (55 ± 5 vs. 35 ± 4, *p* = 0.0498, Figure 2J,L). Using immunofluorescence double‐labeling staining, we further found Sev decreased the expression of FtL in CC‐1‐positive oligodendrocytes of cortex and hippocampus as shown in Figure 2M,N, indicating that Sev caused the iron deficiency in oligodendrocytes. Based on iron's role in growth and development, we speculated that the decrease in iron level in oligodendrocytes may be one of the factors affecting the growth and development of oligodendrocytes.

**FIGURE 3:**
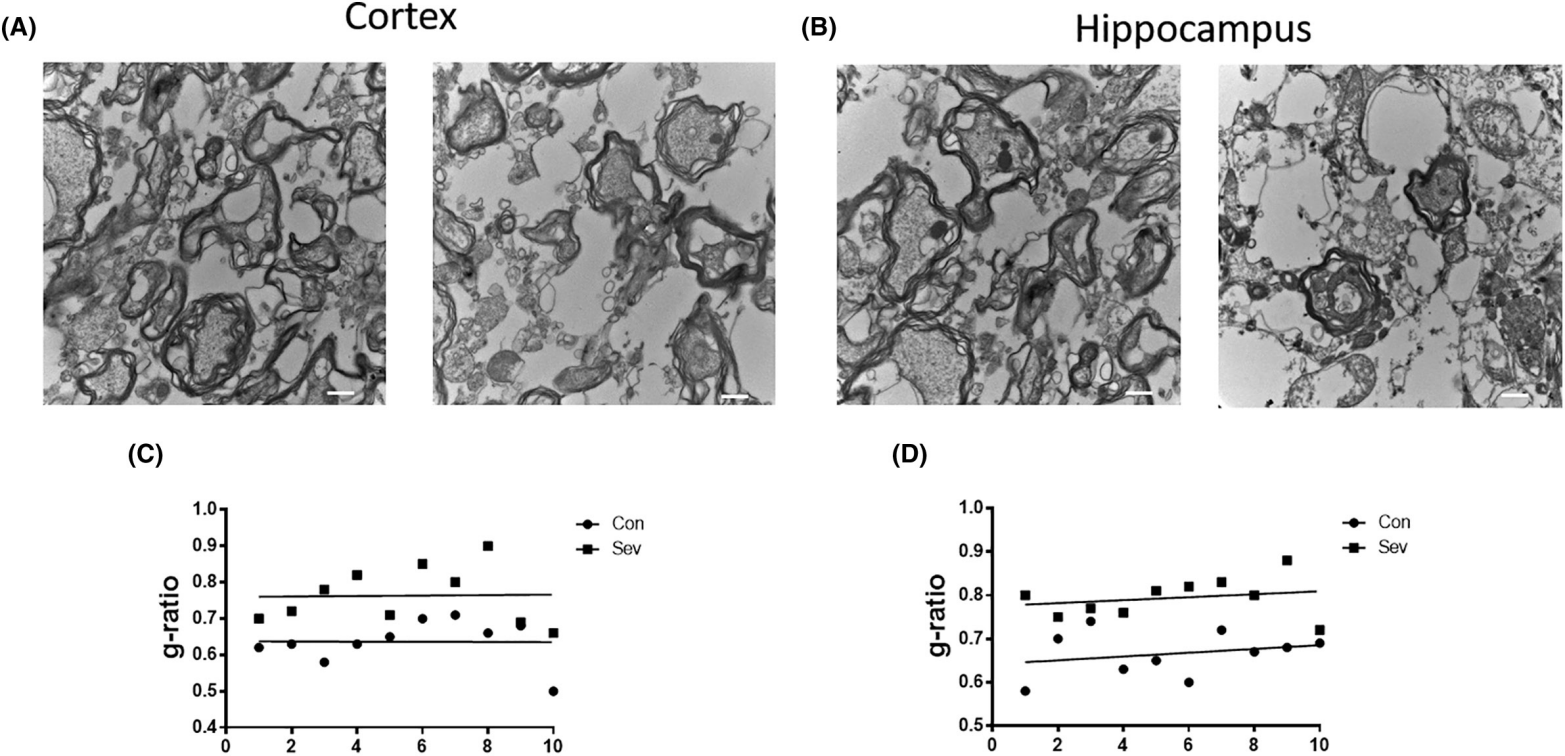
Sev decreased the g ratio of myelin sheath in hippocampus and cortex of infant mice. The infant mice were treated with Sev as described in the ‘Section 2’. Transmission electron microscopy was used to detect the morphological characteristics of myelin sheath in cortex (A) and hippocampus (B) of mice by measuring g ratio (C and D).

In order to further study the effect of Sev on myelin formation, TME was used to detect the myelin morphology of cortical and hippocampal tissues, and the results showed that the g ratio of cortical and hippocampal myelin increased, which meant that Sev caused the reduction of myelin thickness, as shown in Figure 3.

### 3.2 Sev‐induced iron deficiency in neurons

**FIGURE 4:**
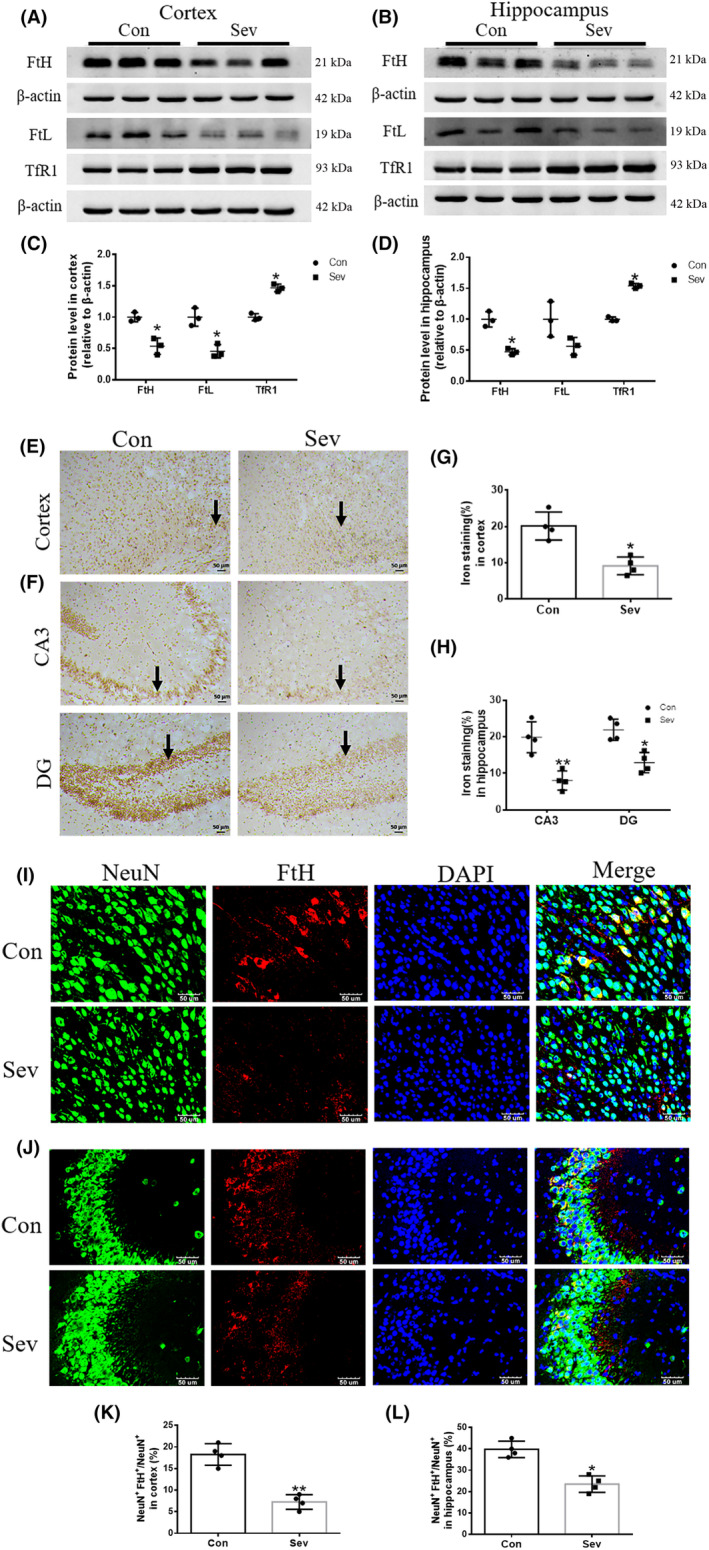
The effects of Sev on expression of FtH, FtL, and TfR1 in cortex and hippocampus or neurons as well as iron staining in cortex and hippocampus. (A–D) The expression of FtH, FtL, and TfR1 in cortex and hippocampus (*n* = 3). (E–H) The iron levels in cortex and hippocampus using Perl's staining (*n* = 4). (I–L) The expression of FtH in neurons from cortex and hippocampus (*n* = 4). Nuclei were counterstained with DAPI. All data are expressed as mean ± SD by Student's *t*‐test. *p < 0.05, **p < 0.01.

Hippocampus and cortex are responsible for cognitive function. In order to evaluate the effect of Sev on the iron homeostasis in cortex and hippocampus tissues, first, we tested the expression of FtH, FtL, and TfR1 in cortex and hippocampus. The results showed that Sev significantly decreased the expression of FtH (*t*_(4)_ = 5.333, *p* = 0.0060, cortex; *t*_(4)_ = 6.770, *p* = 0.0064, hippocampus) and FtL (*t*_(4)_ = 5.233, *p* = 0.0064, cortex) but increased the expression of TfR1(*t*_(4)_ = 9.852, *p* < 0.001, cortex; *t*_(4)_ = 17.12, *p* < 0.001, hippocampus), meaning that Sev caused the iron deficiency in both tissues as shown in Figure 4A–D. Perl's staining also confirmed that Sev indeed lowered the iron levels in cortex and hippocampus as shown in Figure 4E–H (*t*_(4)_=3.525, *p* = 0.0243) and H (*t*_(4)_ = 3.354, *p* = 0.0285, CA3; *t*_(4)_ = 4.097, *p* = 0.0149, DG). Iron levels of whole hippocampus and cortex tissues could not reflect the iron levels difference among all kinds of different nerve cells. Intracellular iron homeostasis is closely regulated by the IRE/IRP system.^20^ Considering the central role of neurons in cognitive function, we used immunofluorescent double staining to evaluate the expression of FtH in neurons of cortex and hippocampus. The results showed that Sev significantly downregulated the expression of FtH in neurons of cortex and hippocampus, indicating that Sev also induced iron deficiency in neurons as shown in Figure 4I–L.

### 3.3 Sev affected the NPCs proliferation in cortex and hippocampus

**FIGURE 5:**
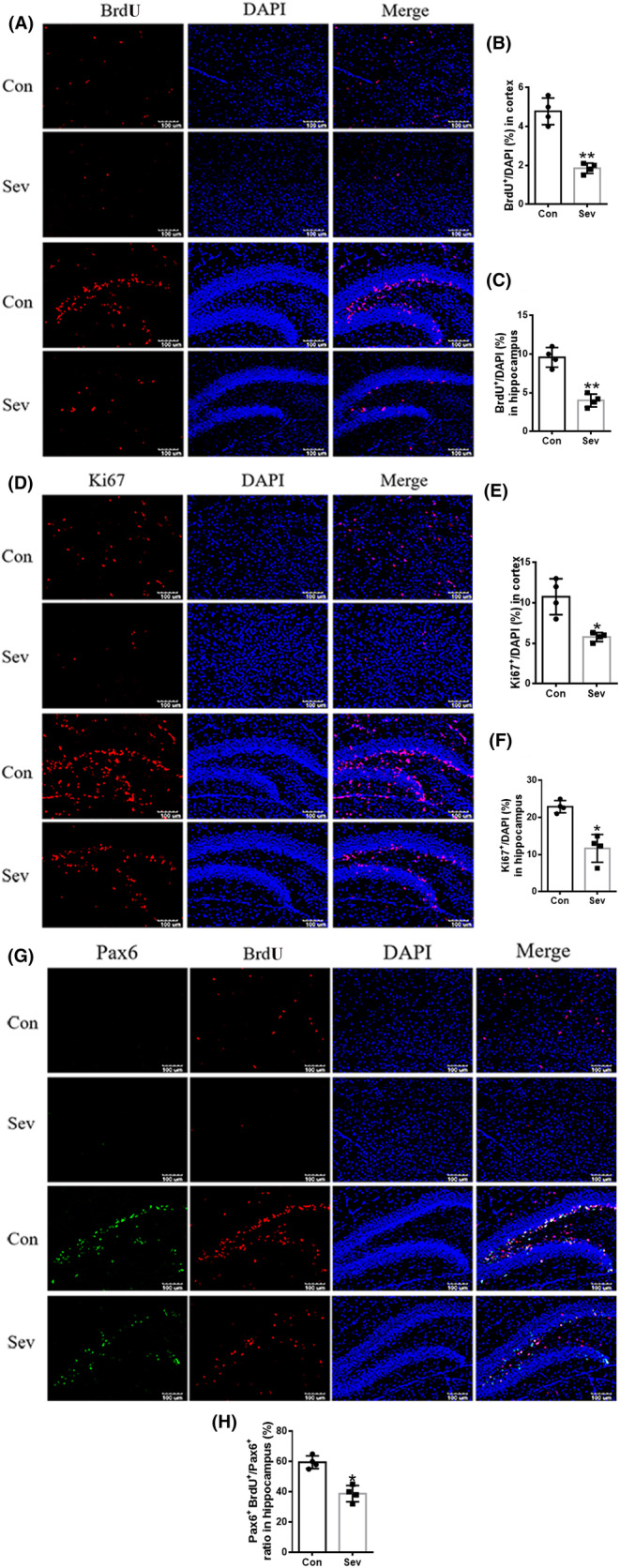
Sev inhibited the DNA synthesis and the NPCs proliferation in cortex and hippocampus. (A) Sev inhibited the cell proliferation using BrdU pulse labeling and followed by staining with BrdU antibody in cortex and hippocampus. (B, C) Percentage of the number of BrdU^+^ cells expressed in the hippocampus and cortex, respectively. (D) The expressions of Ki67 in cortex and hippocampus using immunofluorescence assay. (C) The expression of Ki67 in cortex and hippocampus. (E, F) Percentage of the number of Ki67^+^ cells expressed in the hippocampus and cortex, respectively. (G) Sample images of brain slices with or without Sev treatments, followed by BrdU pulse labeling and immunofluorescence analysis using Pax6 and BrdU antibodies in cortex and hippocampus. Nuclei were counterstained with DAPI. H The cells expressing Pax6 and labeled by BrdU accounted for the percentage of the total cells expressing Pax6 in hippocampus.

Iron was a cofactor of ribonucleotide reductase which played a role in DNA synthesis. Considering the role of cell proliferation of iron and that BrdU incorporation into replicating DNA is a commonly used method to detect cell proliferation, we used BrdU staining to investigate the DNA synthesis induced by Sev in cortex and hippocampus. Our results showed that BrdU‐labeled‐positive cells were significantly reduced in cortex and hippocampus, indicating that Sev significantly inhibited the proliferation in cortex and hippocampus as shown in Figure 5A–C. Ki67 was usually expressed in cells that proliferate vigorously, such as NPCs and NSCs, so it was a biomarker representing cell proliferation.^21^ Our results showed that Sev reduced the number of cells expressing Ki67, indicating that Sev inhibited cell proliferation in cortex and hippocampus as shown in Figure 5D–F. Pax6 was a transcription factor that was expressed in NPCs during neurogenesis specifically in the developing central nervous system.^22^ In order to further illuminate whether Sev inhibited the proliferation of NPCs in cortex and hippocampus, we used immunofluorescence further to test the co‐localization of BrdU and Pax6. We found that Sev caused the BrdU decrease in Pax6+ cells in cortex and hippocampus, indicating that the proliferation of NPCs was inhibited which maybe closely related to iron deficiency as shown in Figure 5G,H.

### 3.4 Sev caused the longer‐term cognitive deficits through decreasing iron levels and inhibited proliferation in NSCs

**FIGURE 6:**
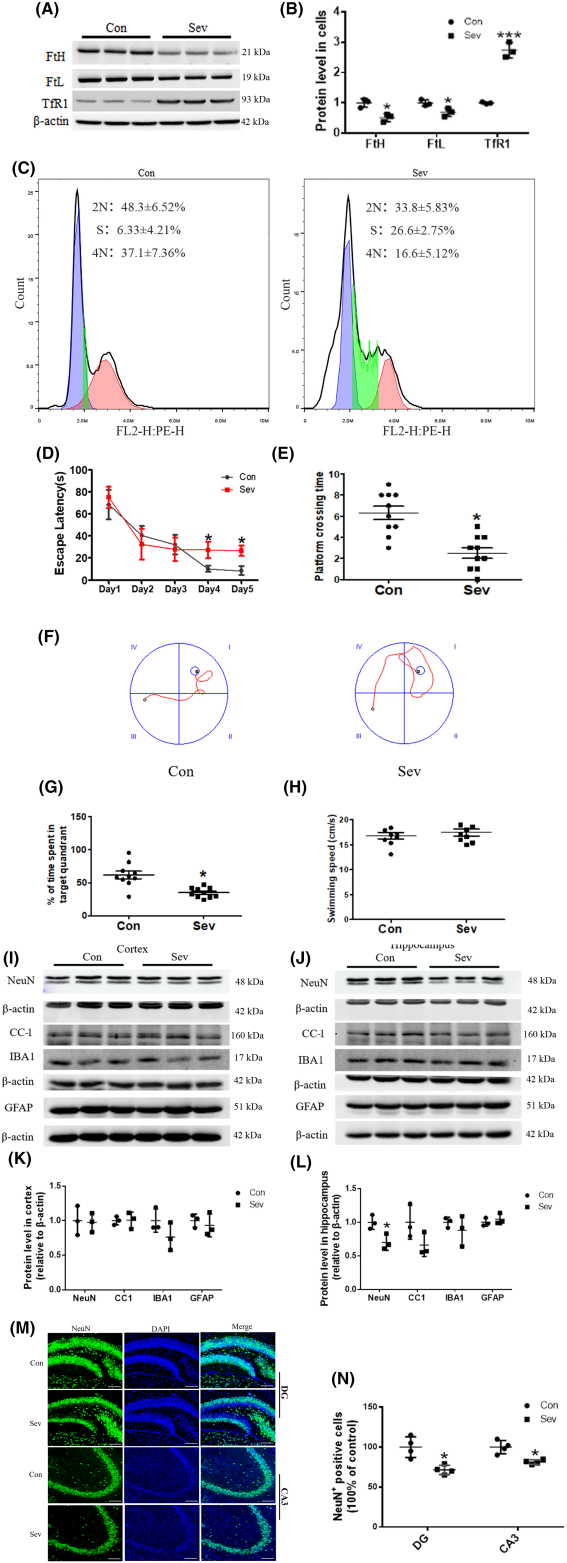
The effects of Sev on iron levels, cell proliferation of NSC, cognitive function, and the number of neurons after 28 days of Sev treatment. (A, B) The expression of FtH, FtL, and TfR1 in NE4C cells (*n* = 3). (C) The effect of Sev on cell proliferation of NE4C cells by flow cytometry. (D–H) The cognitive function test after 28 days of Sev treatment (*n* = 10). (I–L) The effects of Sev on neurons, oligodendrocytes, and microglial cells by detecting biomarker molecule expression using western blot in cortex and hippocampus (*n* = 3). (M, N) The number of mature neurons in DG and CA3 areas (*n* = 4). Nuclei were counterstained with DAPI. Two‐way ANOVA: (D). Mann–Whitney test: (E, G, H). Student's t‐test: (B, K, L, *N*). Data are expressed as mean ± SD, *p < 0.05 and ***p < 0.001.

NPCs are derived from NSCs. In the ventricular zone/subventricular zone (VZ/SVZ) of cortex and DG zone of hippocampus, NSCs /NPCs continuously proliferate and produce new neurons. NE4C cell line was a kind of NSCs from hippocampus of mice. To evaluate the effects of Sev on iron levels and proliferation of NSCs, we investigated the expression of FtH, FtL TfR1, and cell cycle in NE4C cells in vitro. Our results showed that Sev similarly upregulated the expression of TfR1 (*t*_(4)_ = 11.78, *p* < 0.001), downregulated the expression of FtH (*t*_(4)_=4.410, *p* = 0.0116) and FtL (*t*_(4)_ = 3.199, *p* = 0.0329), when the NE4C cells were treated with Sev for 6 h as shown in Figure 6A,B, indicating that Sev also induced the iron deficiency in NE4C cells. Meanwhile, our results showed that the number of cells in 4 N phase of Sev treatment group (16.6 ± 5.12%) was significantly lower than that of the control group (37.1 ± 7.36%, *p* = 0.0163), indicating that Sev inhibited the division and proliferation of NE4C cells through iron deficiency as shown in Figure 6C. It took about 28 days for NSCs to differentiate into other nerve cells.^23^

On the 28th day after anesthesia, we further performed the MWM assay to test the effect of Sev inhibiting NSCs differentiation on cognitive function. The results showed that the mice were still characterized by cognitive deficits as shown in Figure 6D (*F* = 35, *p* = 0.0198, two‐way ANOVA), E (*p* = 0.0483, Mann–Whitney test), F, G (*p* = 0.0385, Mann–Whitney test) and H, indicating that Sev‐induced cognitive impairment maybe had a long‐term effect through affecting NSCs proliferation and differentiation. NeuN, CC‐1, IBA1, and GFAP were widely used as markers of neurons, oligodendrocytes, microglia, and astrocytes, respectively. The expression of these proteins can reflect the functional state of related cells to some extent. To investigate the effects of Sev on these cells after the mice were anesthetized for 28 days, we assayed the expression of these proteins in cortex and hippocampus using western blot. Our results showed that Sev only caused a decrease in NeuN expression (*t*_(4)_=3.182, *p* = 0.0303) and did not influence the expression of other marker proteins of other nerve‐type cells in cortex and hippocampus, indicating that Sev may affect the function of neurons through inhibiting neuron development in hippocampus as shown in Figure 6I–L. To further verify the effect of Sev on neuronal development, we assayed the number of neurons in DG and CA3 areas of hippocampus. The results showed that Sev significantly decreased the number of neurons in hippocampus as shown in Figure 6M,N, indicating that Sev might have damaging effects on neuronal development.

### 3.5 Iron supplementation inhibited the cognitive deficiency induced by Sev

**FIGURE 7:**
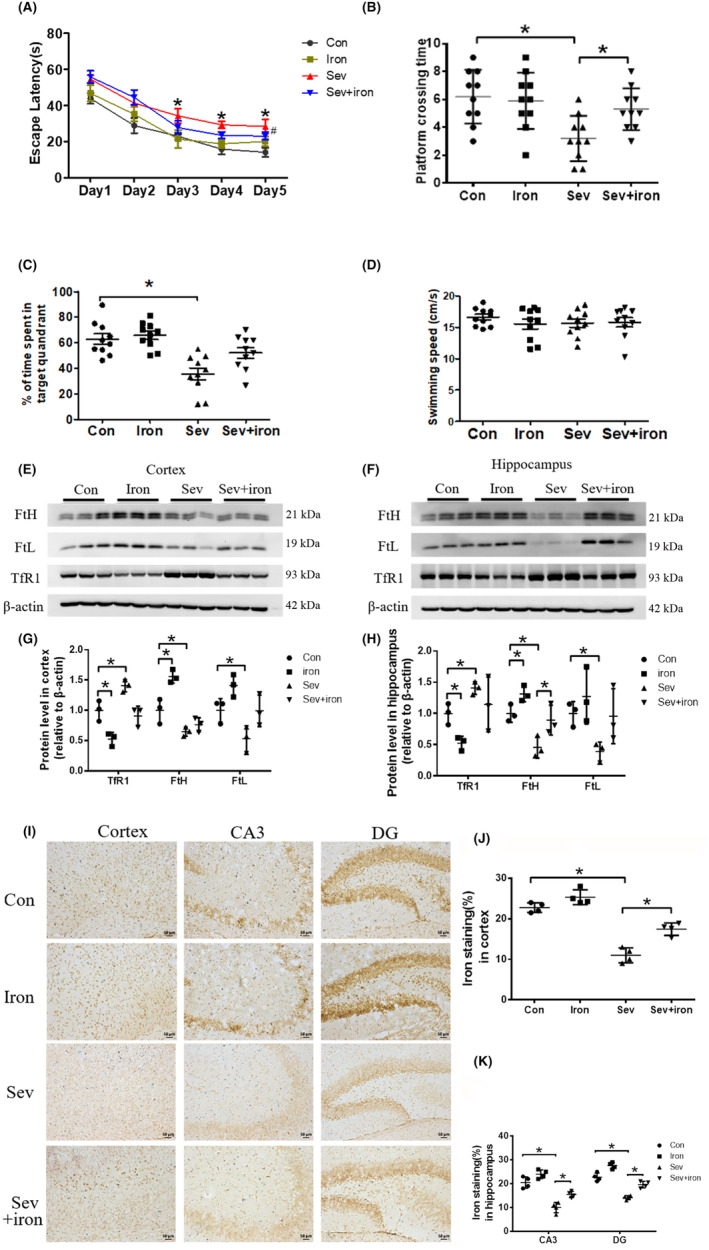
Iron supplementation inhibited the iron deficiency in the brain and rescued the cognitive dysfunction induced by Sev. (A–D) The cognitive function testing by MWM. (E–H) The expression of FtH, FtL, and TfR1 in cortex and hippocampus induced by Sev after iron supplementation. (I–K) The iron levels in cortex and hippocampus induced by Sev after iron supplementation. Data are expressed as mean ± SD (*n* = 10 mice/group for the MWM test; *n* = 3 mice/group for the western blot test). Two‐way ANOVA: (A) Kruskal–Wallis test: (B, C, D). One‐way ANOVA: (G, H, J, K). *p < 0.05.

Given the cognitive deficiency effects of iron deficiency induced by Sev, iron supplementation was performed on the day of pregnancy, and ferrous gluconate solution (0.1 mg/mL) was added to the drinking water of female mice until the 14th day of offspring birth. We further tested and found that iron supplementation dramatically improved the cognitive deficiency in infant mice as shown in Figure 7A (*F*_(3 80)_ = 15.68, *p* = 0.0140, two‐way ANOVA), B (*F*_(3 14)_ = 3.293, *p* = 0.0456, two‐way ANOVA), C (*F*_(3 14)_ = 3.678, *p* = 0.0383, two‐way ANOVA), and D. Meanwhile, iron supplementation also significantly alleviated Sev‐induced iron deficiency and iron metabolism imbalance in cortex and hippocampus as shown in Figure 7E–G (*F*_(3 8)_ = 30.34, *p* < 0.001, TfR1; *F*_(3 8)_ = 26.72, *p* < 0.001, FtH; *F*_(3 8)_ = 8.399, *p* = 0.0075, FtL), H (*F*_(3 8)_ = 6.77, *p* = 0.0138, TfR1; *F*_(3 8)_ = 12.48, *p* = 0.0022, FtH; *F*_(3 8)_ = 3.851, *p* = 0.0486, FtL), I, J (*F*_(3 6)_ = 92, *p* < 0.001), and K (*F*_(3 6)_ = 108, *p* < 0.001, CA3; *F*_(3 6)_ = 181.6, *p* < 0.001, DG). These results suggested that iron supplementation before Sev anesthesia may be an effective strategy to inhibit Sev‐induced cognitive deficiency.

### 3.6 Iron therapy restored iron levels in neurons and oligodendrocytes and rescued the proliferation ability of NPCs and the integrity of myelin sheath

**FIGURE 8:**
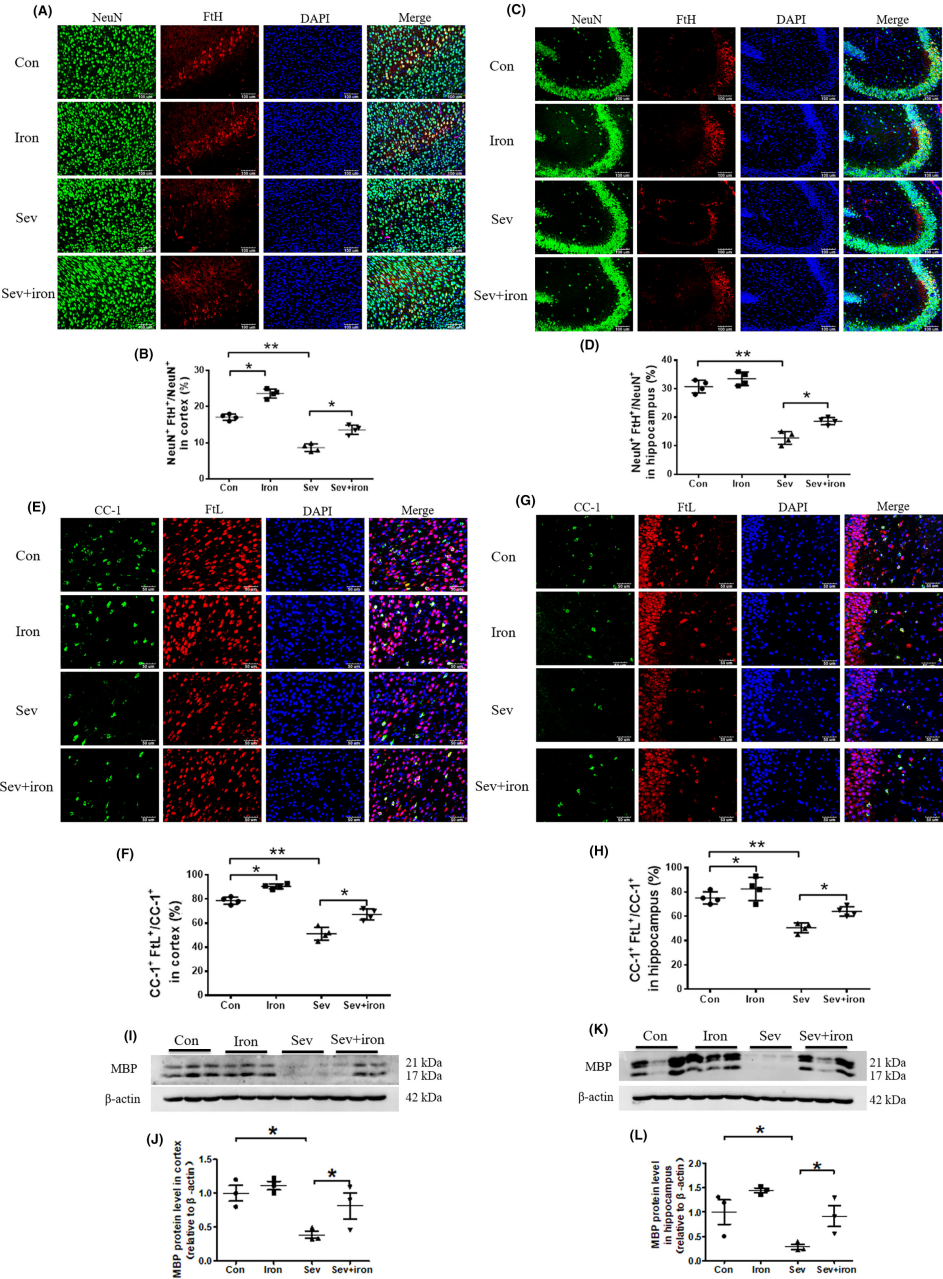
Iron therapy rescued iron deficiency in nerve cells and oligodendrocytes, and myelin formation. (A–D) The FtH expression in neurons (marked by NeuN) of cortex and hippocampus induced by Sev after iron therapy. (E–H) The FtL expression in oligodendrocytes (marked by CC‐1) of cortex and hippocampus induced by Sev after iron therapy. Nuclei were stained with DAPI. (I–L) The MBP expression in cortex and hippocampus induced by Sev after iron therapy. Data are expressed as mean ± SD (*n* = 4). One‐way ANOVA: (G) and (H). *p < 0.05.

In order to further investigate the mechanism of iron supplementation reducing Sev‐induced cognitive deficiency, we used immunofluorescence and western blot to assay the expression of FtH and FtL in neurons and oligodendrocytes and the expression of MBP and Pax6 in cortex and hippocampus. Our results showed that iron therapy significantly compensated for Sev‐induced iron deficiency in mature neurons and oligodendrocytes of cortex and hippocampus as shown in Figure 8A–H. Meanwhile, iron therapy also rescued the decreased expression of MBP in cortex and hippocampus induced by Sev as shown in Figure 8I,J (*F*_(3 6)_ = 5.760, *p* = 0.0336), K and L (*F*_(3 6)_ = 14.02, *p* = 0.0141).

**FIGURE 9:**
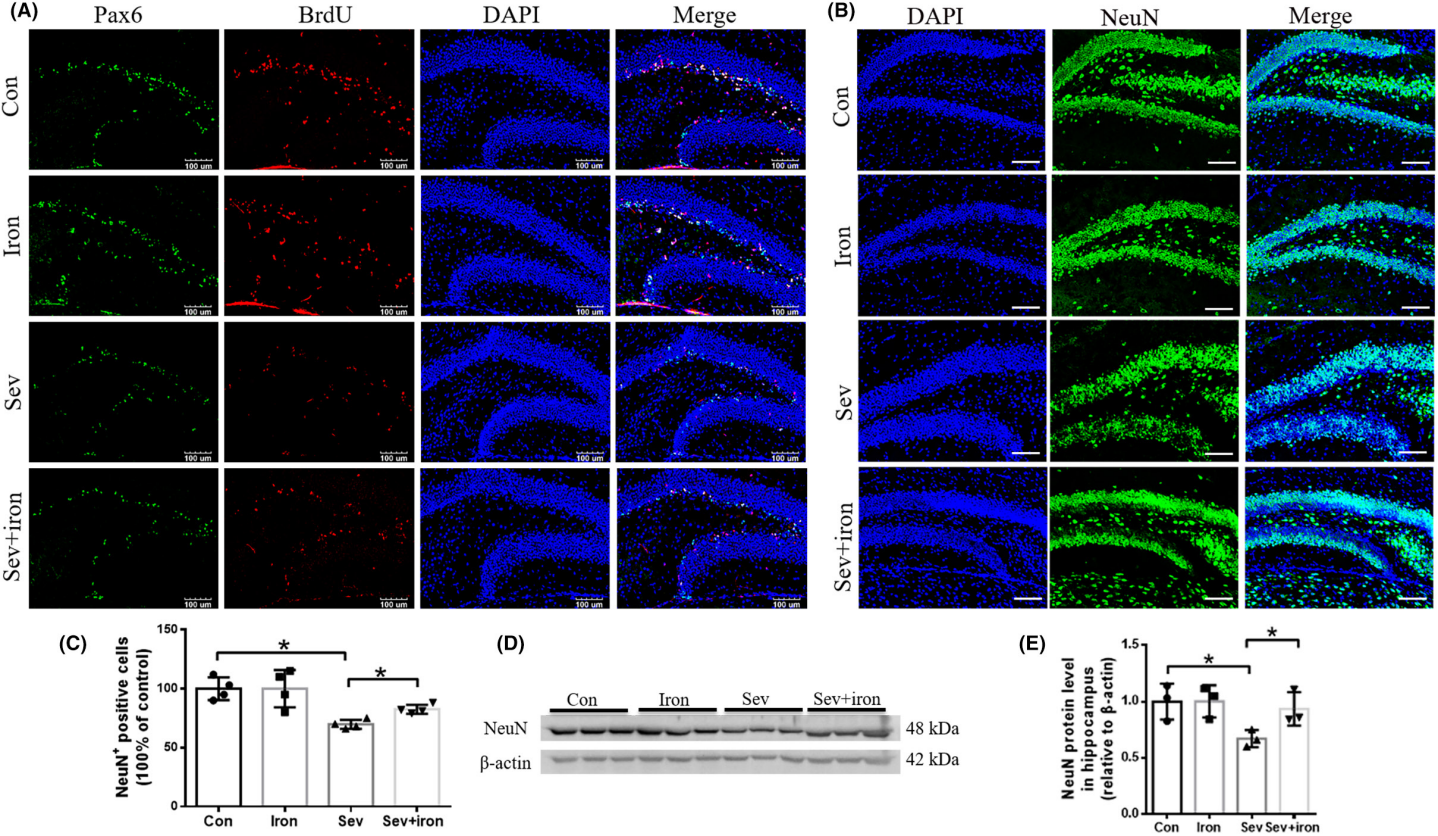
Iron treatment improved the proliferation of progenitor cells after sevoflurane treatment and inhibited the decrease in the number of neurons after 28 days of sevoflurane treatment. (A) The co‐staining of Pax6 and BrdU in hippocampus induced by Sev after iron therapy (*n* = 4). (B, C) the NeuN‐positive cells in hippocampus (*n* = 4). Scale bars: 100 μm. (D, E) the NeuN expression in hippocampus (*n* = 3). Data are expressed as mean ± SD. One‐way ANOVA: (C, E). *p < 0.05.

In order to evaluate the number of NeuN‐positive cells from NSCs/NPCs and the expression of NeuN in the hippocampus after iron supplementation, we further tested the expression of NeuN. As shown in Figure 9A, the results showed that iron supplementation partially restored the reduction in the number of Pax6^+^BrdU^+^ cells induced by Sev in the hippocampus, which indicated that iron supplementation could restore the proliferative capacity of NSCs/NPCs. Furthermore, iron supplementation also partially restored the reduction in the number of NeuN‐positive neurons and decreased NeuN expression induced by Sev (Figure 9B–E), indicating that iron therapy indeed could restore the NSCs/NPCs into mature NeuN‐positive cells in the hippocampus.

## 4 DISCUSSION

Anesthesia was irreplaceable in clinical operations. However, anesthesia could induce neural toxicity causing cognitive dysfunction. For example, postoperative cognitive dysfunction (POCD) was a major clinical issue induced by anesthetics, especially in aged patients.^13^, ^24^ In recent years, the toxic side effects of anesthetics on infants' brains had also been widely concerned,^2^, ^8^, ^25^ but the exact mechanism is not clear. Our previous study had shown that Sev could affect postnatal cognitive function through maternal induction of fetal brain iron metabolism disorder in mice.^6^ In this study, we investigated the mechanism and effect of Sev on the cognitive function with a postnatal 14‐day mouse model to mimic the anesthesia operation of infants in the clinic. Our result showed that escape latency, platform crossing time, and time spent in target quadrant all decreased after the infant mice were exposed to 3% Sev for 6 h, indicating the cognitive function of infant mice appeared deficient (Figure 2A–C). Oligodendrocytes are the main cells of myelination,^26^ and myelinogenesis was closely related to learning and cognitive function.^27^ CC‐1 was the marker molecular of oligodendrocytes.^28^ MBP is one of the most abundant structural proteins of myelin, and its expression levels reflect the status of myelination. To investigate the effect of Sev on myelinogenesis, we tested the expression of MBP and CC‐1 in cortex and hippocampus. Our results showed that Sev dramatically reduced the number of oligodendrocytes and inhibited the myelination, which maybe one of the reasons for cognitive dysfunction in infant mice (Figure 2E–L). Given the important role of iron in the neural system,^29^ we assayed the FtL expression of oligodendrocytes. Our results showed that Sev decreased the FtL expression, indicating that Sev caused the iron deficiency in oligodendrocytes which might be one of the important mechanisms of inhibiting myelination growth and development (Figure 2M,N). The iron in food entered the brain through two barriers, first through the mucosa of the small intestine into the blood, and second, the iron in the blood entered the brain through the blood–brain barrier. Iron transporter 1 (FpN1) played an important role in intestinal iron absorption and was the only iron‐exporting protein known. We found that Svo downregulated FpN1 expression (not shown here), suggesting that Svo reduced intestinal iron absorption which could be one of the causes of iron deficiency in the brain. The above results are consistent with those observed by TME (Figure 3). Hippocampus and cortex tissues were the main brain regions responsible for cognitive ability. Further, we examined the effect of Sev on iron levels of whole hippocampal and cortex tissues. Our results showed that Sev dramatically downregulated the FtH, FtL expression, and upregulated the TfR1 expression in hippocampus and/or cortex, indicating that Sev also caused the iron deficiency in the cortex and hippocampus as shown in Figure 4A–D which was consistent with Perl's iron‐staining results (Figure 4E,F). Considering the role of neurons in cognitive function, we used immunofluorescence double‐labeling staining to investigate the expression of FtH in neurons of cortex and hippocampus. Our results showed that Sev caused FtH decrease in neurons, meaning that Sev induced the iron deficiency of neurons in cortex and hippocampus as shown in Figure 4I–L. Ki67 was one of the markers in cell proliferation and neurogenesis, and Pax6 is expressed highly in NPCs. NPCs play an important role in nerve cell proliferation and neurogenesis. In order to evaluate the effect of Sev on the NPCs proliferation, we tested the DNA synthesis status with BrdU staining, the expression of Ki67, and the co‐staining of Pax6 and BrdU with immunofluorescence assay. We found that iron deficiency induced by Sev suppressed Ki67 expression and DNA replication in NPCs by decreasing BrdU^+^ and Pax6^+^ colocalization in cortex and hippocampus (Figure 5) indicating that Sev inhibited the NPCs proliferation. NSCs/NPCs were the progenitor cells for the growth and development of various nerve cells. In order to investigate the effect of Sev on NSCs, we further tested the expression of FtL, FtH, and TfR1 as well as the cell cycle of NE4C which was a kind of NSC from mice hippocampus in vitro. Interestingly, we found that Sev also caused the iron deficiency of NE4C cells (Figure 6A,B), and cells of 4 N significantly decreased compared with that of the control group (Figure 6C). It took 28 days for NSCs to differentiate into other nerve cells. We continued to monitor the cognitive performance of the infant mice on the 28th day after anesthesia. The results showed that the mice still appeared cognitive deficiency (Figure 6D–H), indicating that iron metabolism dysfunction of NSCs/NPCs induced by Sev affected the number of NSCs/NPCs which was close to the differentiation of many types of nerve cells. Thus, we tested the expression of markers of different types of nerve cells. Our results showed that Sev only affected the expression of NeuN in hippocampus on the 28th after Sev treatment (Figure 6I–L), indicating that Sev may be decreased the number of neurons. Indeed, we further used immunofluorescence to find that the number of neurons in DG and CA3 areas decreased as shown in Figure 6M,N. Since Sev could induce iron deficiency, we administered iron supplementation before anesthesia of mice. Interestingly, our results showed that these damage effects (including MWM, the expression of proteins related to iron metabolism, MBP expression, cell proliferation, and the number of neurons) induced by Sev could be significantly eliminated by iron supplementation pretreatment, indicating that iron therapy could rescue the cognitive impairment, hypomyelination, and inhibition of cell proliferation of NSCs/NPCs induced by Sev, as shown in Figures 7, 8, 9.

In conclusion, these data suggest that Sev‐induced iron deficiency may be one of the important mechanisms of cognitive impairment caused by inhaled anesthetics in infant mice. Iron supplementation before anesthesia is an effective strategy to prevent cognitive impairment caused by Sev in infants.

## AUTHOR CONTRIBUTIONS

Jianhua Zhang and Zhenhua Shi conceived and designed the experiments. Yong Zuo, Jinhong Xie, Xue Zhang, Xiaopeng Liu, and Di Zhang performed the experiments. Yong Zuo and Jinhong Xie completed the statistical analysis of data. Anand Thirupathi and Zhen‐Hua Shi wrote the manuscript.

## CONFLICT OF INTEREST STATEMENT

The authors declare that they have no competing interests.

## Data Availability

All data generated or analyzed during this study are included in this published article and its supplementary information files.
